# Favorable outcome of haploidentical hematopoietic stem cell transplantation in Philadelphia chromosome-positive acute lymphoblastic leukemia: a multicenter study in Southwest China

**DOI:** 10.1186/s13045-015-0186-5

**Published:** 2015-07-26

**Authors:** Li Gao, Cheng Zhang, Lei Gao, Yao Liu, Yi Su, Sanbin Wang, Bin Li, Tonghua Yang, Zhong Yuan, Xi Zhang

**Affiliations:** Department of Hematology, Xinqiao Hospital, Third Military Medical University, Xinqiao Street, Shangpinba District, Chongqing, 400037 China; Department of Hematology, General Hospital of Chengdu Military Region of PLA, Sichuan, China; Department of Hematology, General Hospital of Kunming Military Region of PLA, Yunnan, China; Department of Hematology, Yunnan Provincial Peoples Hospital, Yunnan, China; Department of Hematology, Second Yunnan Provincial Peoples Hospital, Yunnan, China; Department of Hematology, The Affiliated Hospital of Zunyi Medical College, Zunyi, Guizhou China

## Abstract

**Background:**

Since the introduction of tyrosine kinase inhibitors (TKIs) into combination chemotherapy regimens, the majority of newly diagnosed Philadelphia chromosome-positive acute lymphoblastic leukemia (Ph+ ALL) patients have achieved complete remission (CR). However, without allogeneic hematopoietic stem cell transplantation (HSCT), long-term outcomes in adults remain unsatisfactory. Indeed, haploidentical HSCT has become a common treatment for adult patients who lack an HLA-matched donor, though limited data are available on the efficacy of haploidentical HSCT in Ph+ ALL patients.

**Methods:**

We analyzed the clinical outcomes of 82 Ph+ ALL patients who underwent haploidentical HSCT (*n* = 47) or HLA-matched HSCT (*n* = 35). Real-time quantitative reverse transcription polymerase chain reaction (qRT-PCR) was performed to assess BCR-ABL expression. All of the patients were treated with an imatinib-based regimen before undergoing HSCT. Imatinib treatment was resumed in the patients’ posttransplantation following detection of BCR-ABL transcripts.

**Results:**

All of the patients achieved neutrophil and platelet engraftment, with the exception of five patients who died prior to engraftment. Haploidentical HSCT was associated with higher incidences of acute graft-versus-host disease (GVHD) (51.1 vs. 25.7 %, *p* < 0.05) and chronic GVHD (48.9 vs. 25.7 %, *p* < 0.05) compared with HLA-matched HSCT, but there was no difference in the incidence of either grades III–IV acute GVHD or extensive chronic GVHD. The incidence of cytomegalovirus (CMV) infection was significantly higher in the patients treated with haploidentical HSCT than in those treated with HLA-matched HSCT (38.3 vs. 14.3 %, *p* < 0.05). Haploidentical HSCT was associated with a significantly lower relapse rate compared with HLA-matched HSCT (44.8 vs. 19.1 %, *p* < 0.05). There were no differences in non-relapse mortality (NRM), leukemia-free survival (LFS), or overall survival (OS) between the patients who received HLA-matched HSCT and those who underwent haploidentical HSCT.

**Conclusions:**

Our data indicate that the incidence of NRM after HSCT is similar between the patients who receive HLA-matched donor cells and those who receive haploidentical donor cells and that haploidentical HSCT reduces the relapse rate. Haploidentical HSCT represents an encouraging treatment option for Ph+ ALL patients who lack a suitable HLA-matched donor.

## Introduction

Approximately 25–30 % of adults and 3 % of children with acute lymphoblastic leukemia (ALL) express the oncogenic BCR-ABL protein, which results from a 9;22 chromosomal translocation known as the Philadelphia (Ph) chromosome [1]. Ph-positive ALL (Ph+ ALL) is a high-risk subset of ALL that is associated with a lower probability of complete remission (CR) than Ph-negative ALL (Ph− ALL) and that has an extremely poor prognosis [2]. Most studies have found that Ph+ ALL patients have a median survival time of 6–12 months [3, 4]. Although the introduction of tyrosine kinase inhibitors (TKIs) into the standard combination chemotherapy regimens for newly diagnosed Ph+ ALL allows over 95 % of patients to achieve CR [5, 6], Ph+ ALL remains an unfavorable prognostic subgroup with an unacceptably high relapse rate. In the UKALLXII/ECOG2993 trial, the addition of imatinib to the chemotherapeutic cocktail in the absence of myeloablative allogeneic hematopoietic stem cell transplantation (allo-HSCT) did not result in a significant survival benefit [7]. Allo-HSCT during the first CR (CR1) remains the optimal curative treatment for Ph+ ALL to date [3]. The most therapeutically favorable treatment for Ph+ ALL is allo-HSCT from an HLA-identical sibling donor, but approximately 70 % of patients lack a suitable sibling donor [8]. In China, matched unrelated donors [9] are found for only 20 % of patients who lack a suitable sibling donor, and umbilical cord blood units are not always suitable for adult transplantation because they contain insufficient numbers of cells. However, nearly all patients have at least one HLA-haploidentical matched family member. Recent comparative studies have shown that acute leukemia patients who receive either haplotransplantation or HLA-matched transplantation have similar outcomes [10, 11], but the efficacy and safety of haplotransplantation in Ph+ ALL patients have not been examined. Our current study compares the outcomes of patients who have been diagnosed with Ph+ ALL and treated with HSCT using cells from either haploidentical donors or matched donors.

## Materials and methods

### Patients

We analyzed the clinical data of 82 patients with Ph+ ALL who underwent either HLA-matched HSCT or haploidentical HSCT at one of five hospitals in southwest China between July 2007 and April 2011. The diagnosis of Ph+ ALL was based on the diagnostic criteria of the World Health Organization (WHO). The BCR-ABL fusion gene was detected by real-time quantitative polymerase chain reaction (PCR). The study was reviewed and approved by the ethics committees of the participating institutions. All of the patients included in this study provided informed consent in accordance with the Declaration of Helsinki.

### Conditioning regimen and imatinib therapy

The patients undergoing HLA-matched HSCT were conditioned with 9.0–10.5 Gy total-body irradiation (TBI) on days −5 and −4 and 60 mg/kg/day intravenous cyclophosphamide (CY) on days −3 and −2. The patients undergoing haploidentical HSCT were conditioned with 9.0–10.5 Gy TBI on days −7 and −6, 6 g/m^2^/day intravenous arabinosylcytosine (Ara-C) on days −5 to −3, 45 mg/kg/day intravenous CY on days −3 to −2, and 2.5 mg/kg/day intravenous anti-thymocyte globulin (ATG) (Sanofi, SangStat, Lyon, France) on days −5 to −2.

All of the patients received imatinib prior to transplantation. Imatinib was administered posttransplantation only if BCR-ABL transcripts were detectable by real-time quantitative PCR and if the patients could tolerate oral imatinib without developing gut graft-versus-host disease (GVHD) or life-threatening infection.

### Donors, stem cell mobilization, and stem cell collection

Peripheral blood (PB) and bone marrow (BM) cells were collected from the donors using standard mobilization protocols. Granulocyte colony stimulating factor (G-CSF) (5 μg/kg/day; Filgrastim, Kirin Pharma Co., Ltd., Japan) was administered to the donors for 5–6 days to mobilize stem cells in the BM (G-BM) and PB (G-PB). Starting on the fifth day of G-CSF administration (day 1), G-PB cells were harvested by large-volume leukapheresis using a Fenwal CS3000 apparatus (Fenwal, Deerfield, IL, USA). If the number of mononuclear cells (MNCs) or CD34+ cells harvested was not sufficient for HLA-matched HSCT, additional G-PB cells were harvested on day 2. On day 2, G-BM cells were harvested for haploidentical HSCT. The goal was to collect at least 4 × 10^8^ MNCs and 2 × 10^6^ CD34+ cells per kilogram of the recipient’s body weight.

### GVHD prophylaxis and management

The patients who received HLA-matched HSCT were treated posttransplantation with mycophenolate mofetil (MMF), cyclosporin A (CsA), and methotrexate (MTX) [12]. A total of 500 mg (15 mg/kg for pediatric patients) of MMF (Roche Pharmaceutical, Ltd., Switzerland) were administered orally every 12 h from day 1 until day 30. Continuous intravenous CsA (2.5 mg/kg/day) was administered starting on day −1 and was continued until the patient was able to tolerate oral medication. Next, 2.5 mg/kg CsA was administered orally twice per day to achieve a blood concentration of 200–300 ng/mL for 90 days. The dose of CsA was then gradually reduced. In total, 15 mg/m^2^ MTX was administered intravenously on day 1, and 10 mg/m^2^ MTX was administered on days 3 and 6. The patients who received haploidentical HSCT were treated posttransplantation with ATG, MMF, CsA, and MTX. In addition to the standard treatment with ATG, MMF was administered from day −7 until day 90. Continuous intravenous CsA (1.5 mg/kg/day) was started on day −7, and the dose was increased to 2.5 mg/kg/day on day −1, and it was replaced with oral medication. Over the next 150–180 days, the dose of CsA was gradually reduced. A total of 15 mg/m^2^ MTX were administered on day 1, and 10 mg/m^2^ MTX were administered on days 3, 6, and 11.

The patients who developed GVHD were treated with 1–2 mg/kg/day methylprednisolone or prednisolone and 2.5 mg/kg/day CsA. The patients who developed steroid-refractory GVHD were treated with second-line immunosuppressive therapies, such as MMF, tacrolimus (FK506, Astellas, Japan), or anti-CD25 monoclonal antibodies (Novartis Pharma, Ltd., Switzerland).

### Evaluation and definitions

The degree of hematopoietic chimerism was determined using a PCR-based assay that detects short tandem repeats in DNA. Full donor chimerism was defined by the detection of 95 % or more donor cells in whole-blood samples [13]. Disease relapse was defined on the basis of morphology, detection of the BCR-ABL fusion gene, or evidence of leukemic cells in either the BM or other extramedullary organs. Leukemia-free survival (LFS) was defined as the shortest interval between HSCT and relapse or non-relapse mortality (NRM) or the last follow-up. Transplantation-related toxicity (TRT) was evaluated using standard criteria established by the National Cancer Institute (NCIC; www.ecog.org/general/ctc). Damage to the patients’ organs that was due to either GVHD or infectious complications was excluded from this analysis.

### Statistical analysis

Overall survival (OS) and LFS were estimated using the Kaplan-Meier method. GVHD, disease relapse, and NRM rates were estimated using cumulative incidence analysis. For GVHD, death without an event was a competing risk. NRM and relapse were considered to be mutually competing risks. Univariate analysis was conducted using Cox regression. Multivariate analysis was performed using the Cox proportional regression model. SPSS version 16.0 statistical software was used for Kaplan-Meier, univariate, and multiple regression analyses. R software version 2.15.2 was used for competing risk analysis for GVHD, NRM, and relapse.

## Results

### Characteristics of patients and donors

The demographic characteristics and relevant transplantation data for the patients who were included in this study are shown in Table 1. The characteristics of the patients who received either HLA-matched HSCT or haploidentical HSCT were not significantly different. Many of the patients achieved CR and were BCR-ABL fusion gene-negative after several cycles of chemotherapy because all of the patients received imatinib in combination with standard chemotherapy prior to transplantation. In the HLA-matched HSCT group, seven patients were BCR-ABL fusion gene-positive, and the BCR-ABL gene expression rate before HSCT was 2.95 %. In the haploidentical HSCT group, ten patients were BCR-ABL fusion gene-positive, and the BCR-ABL gene expression rate before HSCT was 2.42 %. All 17 patients were BCR-ABL fusion gene-negative posttransplantation.

**Table 1:** Characteristics of patients and donors

Characteristic	HLA-matched HSCT	Haploidentical HSCT	*P*
	(*n* = 35)	(*n* = 47)	
Age, years, median (range)	35(5–52)	35(8–50)	0.679
Males/females, *n*	22/13	24/23	0.287
WBC count at diagnose × 10^9^, median (range)	45.0(1.4–199.2)	36.3 (1.6–274.4)	0.147
CNS involvement			
Yes/no	3/32	6/41	0.548
Disease status at transplantation			
CR1	26	37	0.584
>CR1	9	10	
BCR-ABL at transplantation			
Negative/positive	28/7	37/10	0.888
Average mos. from diagnosis to transplant (range)	9.0(3–47)	7.0(2–19)	0.104
Cycles of prior chemotherapy (range)	5.3(2–22)	4.6(2–13)	0.411
ABO match, no. (%)			0.224
Matched	12	27	
Minor mismatched	8	7	
Major mismatched	11	9	
Major and minor mismatched	4	4	
Donor-recipient sex match			0.323
Male-male	10	16	
Male-female	9	18	
Female-male	12	8	
Female-female	4	5	

### Engraftment and chimerism

The numbers of MNCs and CD34+ cells are shown in Table 2. Excluding the two patients who received HLA-matched HSCT and the three patients who received haploidentical HSCT who died prior to engraftment, 77 patients achieved engraftment of both neutrophils and platelets. The median time to achieve an absolute neutrophil count (ANC) >0.5 × 10^9^ cells/L were 14 days (range 9–54 days) in the patients who received HLA-matched HSCT and 15 days (range 10–22 days) in those who received haploidentical HSCT (*p* = 0.225). The median time until platelet engraftment totaled >20 × 10^9^ cells/L were 14 days (range 9–210 days) in the patients who received HLA-matched HSCT and 15 days (range 10–33 days) in those who received haploidentical HSCT (*p* = 0.134). All of the surviving patients had complete donor chimerism by 60 days after transplantation.

**Table 2:** Cell yield (cells/kg recipient body weight) given in average values with minimum and maximum in parentheses

	MNC(×10^8^)	CD34+ (×10^6^)
G-PBSCs graft		
For matched HSCT	9.48(4.27–20.5)	6.48(1.39–31.60)
G-PBSCs graft		
For haploidentical HSCT	7.55(2.5–19.71)	4.92(1.03–12.71)
G-BM		
For haploidentical HSCT	4.41(1.6–8.46)	1.56(0.54–3.64)

### GVHD and toxicity

**Fig. 1:**
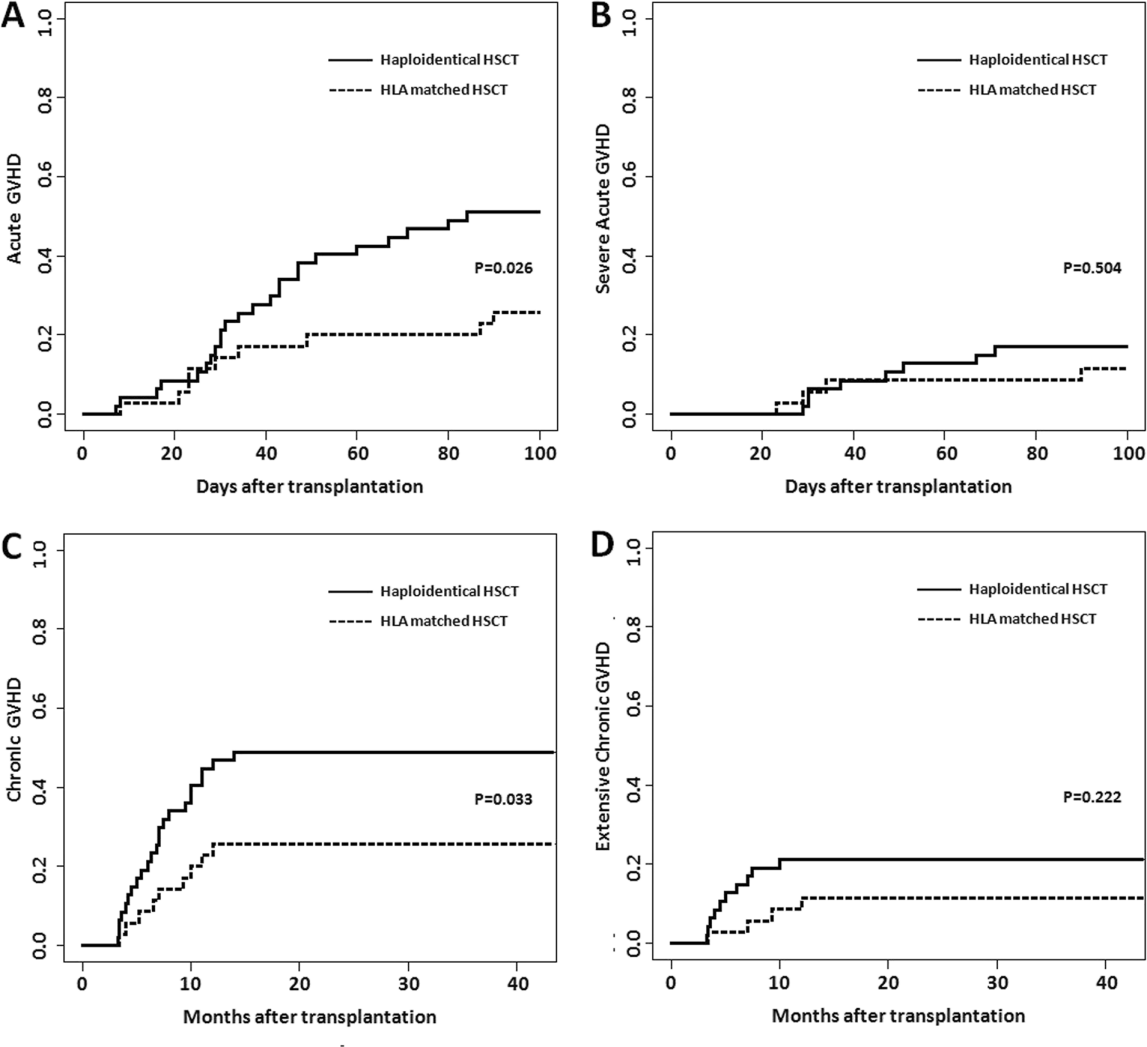
Cumulative incidence of GVHD. **a** Acute GVHD, **b** severe acute GVHD, **c** chronic GVHD, and **d** extensive chronic GVHD

The cumulative incidence of acute GVHD (aGVHD) among the patients who received haploidentical HSCT (24 patients, 51.1 %; 95 % (CI), 35.8–64.4 %) was significantly higher than that among those who received HLA-matched HSCT (9 patients, 25.7 %; 95 % (CI), 12.6–41.1 %) (*p* = 0.026, Fig. 1a). There was no difference in the incidence of grades III–IV aGVHD between the patients who received HLA-matched HSCT (11.4 %; 95 % (CI), 3.5–24.5 %) and those who received haploidentical HSCT (17.0 %; 95 % (CI), 7.8–29.1 %) (*p* = 0.504, Fig. 1b). The 2-year cumulative incidence of chronic GVHD (cGVHD) was higher among the patients who received haploidentical HSCT (23 patients, 48.9 %, 95 % (CI), 33.8–62.4 %) than among those who received HLA-matched HSCT (9 patients, 25.7 %, 95 % (CI), 12.6–41.1 %) (*p* = 0.033, Fig. 1c). At the time of analysis, four of the patients who received HLA-matched HSCT showed signs of extensive cGVHD (cumulative incidence, 11.4 %; 95 % (CI), 3.5–24.5 %), whereas ten of the patients who received haploidentical HSCT showed such signs (cumulative incidence, 21.3 %; 95 % (CI), 10.9–34.0 %) (*p* = 0.222, Fig. 1d).

The toxicities of the regimens are summarized in Table 3. There was no hepatic venous occlusive disease (HVOD) in either group. The incidences of creatinine elevation, hemorrhagic cystitis, and heart dysfunction were very low in both groups. The incidence of CMV infection was significantly higher among the patients who received haploidentical HSCT than among those who received HLA-matched HSCT. Among the patients who received haploidentical HSCT, three developed CMV pneumonia and one developed CMV enteritis. Two of these patients ultimately died of CMV pneumonia. The incidences of other toxicities were not significantly different between the two groups.

**Table 3:** Toxicities by HSCT and infection

Toxicity	HLA-matched HSCT	Haploidentical HSCT	*P*
Hepatic			
Transaminase elevation	8(22.9 %)	14(29.8 %)	0.484
Bilirubin elevation	3(8.6 %)	9(19.1 %)	0.180
Gastrointestinal tract			
Diarrhea	25(71.4 %)	33(70.2)	0.905
Nausea and vomiting	17(48.6 %)	32(68.1 %)	0.075
Mucositis	13(37.1 %)	21(44.7 %)	0.493
Alimentary tract hemorrhage	2(5.7 %)	6(12.8)	0.287
Urinary tract/kidney			
Creatinine elevation	0	1(2.1)	0.385
Hemorrhagic cystitis	1(2.9 %)	1	0.832
Infection			
Bacterial infection	23(65.7 %)	32(68.1 %)	0.821
Fungal infection	4(11.4 %)	8(17.0 %)	0.479
CMV infection	5(14.3 %)	18(38.3 %)	0.017
Cardiovascular			
Heart dysfunction	1	0	0.244

*CMV* cytomegalovirus

### Relapse, NRM, OS, and LFS

**Fig. 2:**
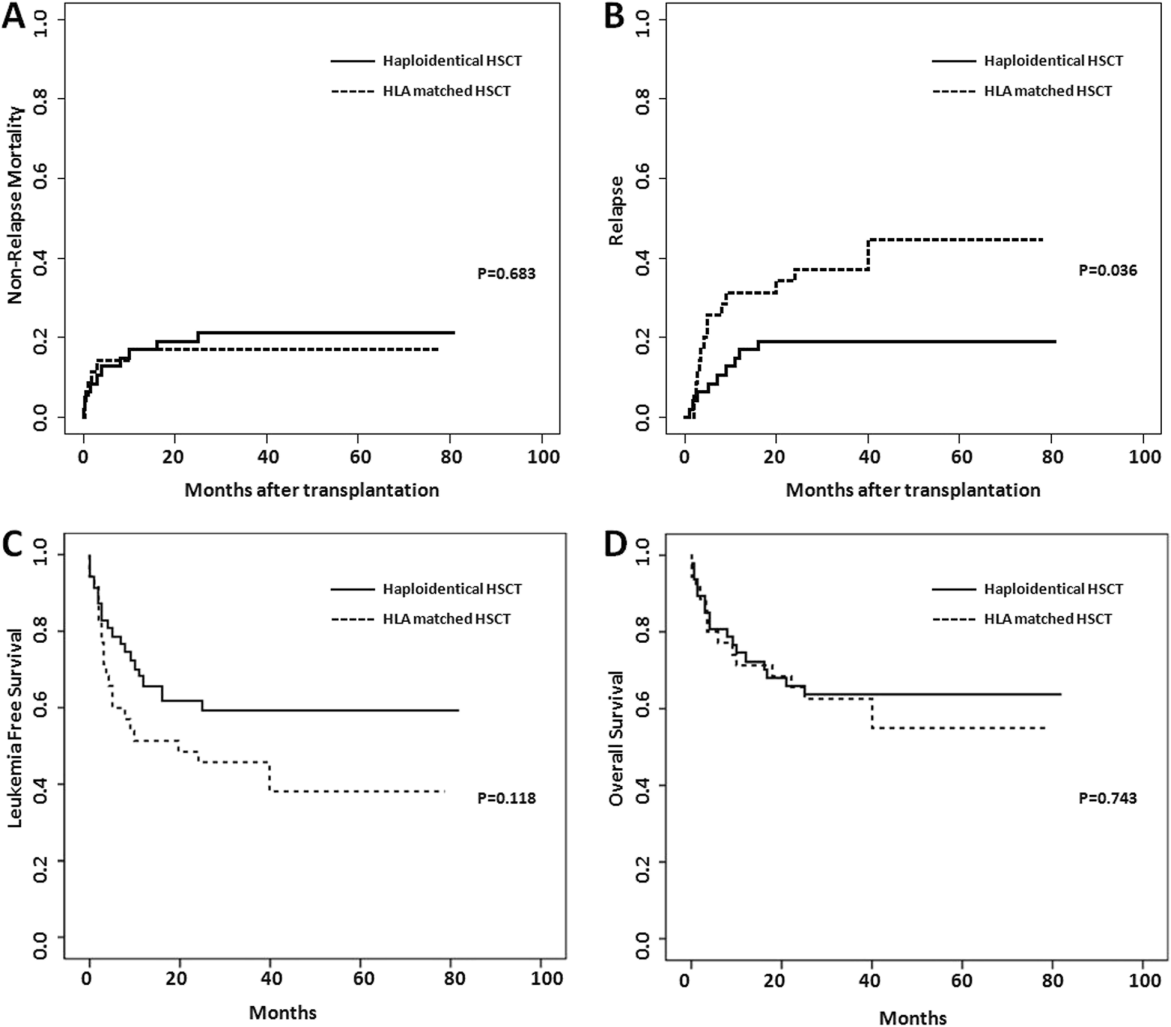
**a** Cumulative incidence of NRM. **b** Cumulative incidence of relapse. **c** Kaplan-Meier estimate of LFS. **d** Kaplan-Meier estimate of OS

Five of the transplant patients died before engraftment was achieved, and 77 achieved molecular CR after transplantation. The patients who received HLA-matched HSCT were monitored for a median time of 26 months (range 0.2–78 months), and those who received haploidentical HSCT were monitored for a median time of 31 months (range 0–81 months). Relapse was defined as molecular relapse, hematological relapse, or extramedullary leukemia relapse. The incidence of relapse was higher among the patients who received HLA-matched HSCT (14 patients) than among those who received haploidentical HSCT (9 patients) (cumulative incidence, 44.8 vs. 19.1 %; 95 % (CI), 23.8–63.7 % vs. 9.3–31.6 %) (*p* = 0.036, Fig. 2a). The median time until relapse was 4.6 months in the patients who received HLA-matched HSCT and 7.0 months in those who received haploidentical HSCT. After recurrence, three patients in the HLA-matched HSCT group and two in the haploidentical HSCT group ceased treatment and died a few weeks later. The other patients who experienced relapse received imatinib treatment, and several of them also received chemotherapy, intrathecal chemotherapy, DLI, or biotherapy. The remaining six patients in the HLA-matched HSCT group and two in the haploidentical HSCT group are still alive; three of them have central nervous system leukemia (CNSL), and five are BCR-ABL fusion gene-positive. Haploidentical HSCT was associated with a significantly lower risk of relapse, as determined by univariate analysis (Table 4) and confirmed by multivariate analysis (hazard ratio (HR) 0.413; 95 % (CI), 0.178–0.958) (*p* = 0.039). However, >CR1 was a risk factor for recurrence, as determined by univariate (Table 4) and multivariate analysis (HR 2.694; 95 % (CI), 1.121–6.475) (*p* = 0.027). The patients who had cGVHD appeared to have a lower risk of relapse by univariate analysis but not by multivariate analysis. Other factors, such as age, white blood cell (WBC) count at diagnosis, and the presence of aGVHD, did not affect the risk of relapse.

**Table 4:** Results of univariate analysis of RE, LFS and OS

	RE		LFS		OS	
Variable	HR(95 % CI)	*P*	HR(95 % CI)	*P*	HR(95 % CI)	*P*
Age						
<40 years	1(Reference)		1(Reference)		1(Reference)	
≥40 years	0.752(0.279–2.026)	0.573	0.932(0.454–1.913)	0.849	0.954(0.427–2.314)	0.910
HLA disparity						
Identical HSCT	1(Reference)		1(Reference)		1(Reference)	
Haploidentical HSCT	0.399(0.173–0.924)	0.032	0.609(0.325–1.143)	0.123	0.889(0.438–1.803)	0.744
Duration from diagnosis to SCT						
<180 days	1(Reference)		1(Reference)		1(Reference)	
≥180 days	1.775(0.783–4.025)	0.169	1.389(0.740–2.609)	0.306	0.915(0.444–1.886)	0.810
Disease status before transplantation						
CR1	1(Reference)		1(Reference)		1(Reference)	
>CR1	3.289(1.429–7.569)	0.005	2.279(1.179–4.408)	0.014	1.711(0.804–3.642)	0.164
WBC at diagnosis						
<30 × 10^9^/L	1(Reference)		1(Reference)		1(Reference)	
≥30 × 10^9^/L	2.272(1–5.165)	0.05	1.842(0.976–3.478)	0.06	1.590(0.778–3.249)	0.204
Acute GVHD						
Yes	1(Reference)		1(Reference)		1(Reference)	
No	0.562(0.235–1.366)	0.204	0.814(0.427–1.552)	0.532	1.208(0.595–2.451)	0.601
Chronic GVHD						
Yes	1(Reference)		1(Reference)		1(Reference)	
No	0.5(0.253–0.988)	0.046	0.570(0.241–1.346)	0.2	0.584(0.275–1.242)	0.162
CMV						
Positive	1(Reference)		1(Reference)		1(Reference)	
Negative	0.511(0.173–1.506)	0.223	1.087(0.55–2.148)	0.81	1.508(0.721–3.155)	1.508

There was no significant difference in the incidence of NRM between the patients who received HLA-matched HSCT (cumulative incidence, six patients, 17.1 %; 95 % (CI), 6.8–31.4 %) and those who received haploidentical HSCT (cumulative incidence, ten patients, 21.3 %; 95 % (CI), 10.9–34.1 %) (*p* = 0.683, Fig. 2b). The incidences of NRM were 11.8 and 21.5 % in the BCR-ABL-positive and BCR-ABL-negative patients, respectively.

The Kaplan-Meier estimate of the 2-year OS of the patients who received haploidentical HSCT was 63.8 %, which was similar to that of the patients who received HLA-matched HSCT (62.6 %) (*p* = 0.743, Fig. 2c). Univariate analysis revealed that sex, the WBC count, the presence of GVHD, and HLA disparity did not significantly influence the patients’ OS (Table 4). At 2 years, the LFS of the patients who received haploidentical HSCT was higher than that of the patients who received HLA-matched HSCT (59.5 vs. 45.7 %) (*p* = 0.118), although this difference was not statistically significant (Fig. 2d). >CR1 at the time of transplantation significantly influenced LFS, as determined by univariate analysis, but this difference was not confirmed by multivariate analysis (HR 1.895, 95 % (CI), 0.955–3.761) (*p* = 0.068). The cumulative 2-year OS rates were 52.9 and 69.2 % in the BCR-ABL-positive and BCR-ABL-negative patients, respectively (*p* = 0.325). At 2 years, the LFS rates were 41.2 % for the BCR-ABL-positive patients and 58.5 % for the BCR-ABL-negative patients (*p* = 0.229). Although there were no significant differences in OS or PFS due to the limited number of patients included in this study, the BCR-ABL-negative patients tended to have worse outcomes.

## Discussion

Ph+ ALL is an aggressive disease with a poor prognosis. Despite an initial favorable response to treatment, the long-term outcome is unsatisfactory in adults when TKI/chemotherapy combinations are used without allo-HSCT. This unfavorable prognosis might justify the use of alternative conditioning options and higher-risk donors, such as mismatched unrelated donors or haploidentical donors or umbilical cord blood-derived cells. It is difficult to assess the balance between a greater risk of treatment-related mortality (TRM) and an increased risk of relapse. In recent years, considerable progress has been made in haploidentical HSCT, and the clinical outcomes as well as the health-related quality of life (HRQoL) of patients receiving this treatment have been reported to be comparable to those of patients receiving HLA-matched HSCT [14, 15]. Although haploidentical HSCT has become a common treatment in adult patients who lack a HLA-matched donor, data on its efficacy in Ph+ ALL are limited. We conducted multicenter analysis of 82 Ph+ ALL patients in southwest China undergoing either HLA-matched HSCT or haploidentical HSCT to examine the effect of haploidentical HSCT on the outcome of Ph+ ALL.

In recent studies, historical comparisons of patients from the pre-TKI era have shown that patients who are treated with TKIs plus chemotherapy have significantly improved CR rates and prolonged LFS [16–18]. Moreover, the pretransplantation use of imatinib improves the outcome of allo-HSCT and patients’ candidacy of HSCT [19, 20]. Based on these data, there is now no rationale for omitting TKIs from induction treatment; accordingly, all of the patients in our study received imatinib in combination with standard chemotherapy before transplantation.

Certain studies have found that patients who receive haploidentical HSCT experience significantly delayed myeloid and platelet recoveries compared with those receiving HLA-matched HSCT [14, 21]. However, we found no difference in engraftment between the patients who received haploidentical HSCT and those who underwent HLA-matched HSCT. Differences between our study and these previous studies include the use of PBMCs plus BM, the lack of in vitro T cell depletion, and the use of greater numbers of MNCs and CD34+ cells in our study. Different studies have obtained different results regarding the prevalence of GVHD. Luo et al. have reported that grades II–IV aGVHD and severe aGVHD are significantly more frequent in patients undergoing haploidentical HSCT compared with those receiving matched sibling donor HSCT but that the incidence of cGVHD is not significantly affected by donor type [14]. In other studies, the incidence of GVHD has been reported to be similar between patients who have received haploidentical HSCT and those who have undergone matched sibling donor HSCT [21]. Our results showed that the incidences of both aGVHD and cGVHD were higher in patients who received haploidentical HSCT compared with those who received HLA-matched HSCT, but there were no differences between the two patient groups with regard to the incidences of severe aGVHD and extensive cGVHD. Modern treatment strategies have reduced complications in patients receiving haploidentical HSCT, but Wang et al. reported that the incidence of CMV viremia increased to 64 % among patients who underwent haploidentical HSCT [22]. In our study, the incidence of CMV viremia in patients who received haploidentical HSCT was higher than that in those receiving HLA-matched HSCT. This discrepancy was associated with the intensified GVHD prophylactic strategy used in haploidentical HSCT [23]. Apart from the incidence of CMV infection, there were no differences in the toxicities of the regimens between the two groups, and no patients in either group experienced HVOD.

The most important finding of this study is that the patients who received haploidentical HSCT had a significantly lower incidence of relapse than those who received HLA-matched HSCT. The lower incidence of relapse among the patients who received haploidentical HSCT may have been due to the graft-versus-leukemia (GVL) activity of haploidentical HSCT. We observed a significantly lower rate of relapse in the patients with cGVHD compared with those without this disease, in agreement with previously published observations [10, 24, 25]. cGVHD has been associated with a putative GVL effect, and several studies have shown that it contributes significantly to the eradication of MRD [10, 24, 25]. The conditioning regimen of haploidentical HSCT is more intensive than that of HLA-matched HSCT, which is related to the lower rate of relapse in haploidentical HSCT. Previous studies have confirmed a relationship between chemotherapeutic regimen intensity and disease recurrence risk. Bredeson et al. [26] compared patients received FB with those received BuCy, finding that the relapse rate was higher after FB treatment than after BuCy treatment. Bachanova et al. [27] have confirmed that a less intense regimen may not be sufficient to eliminate residual detectable leukemia.

In our study, the relapse rates were 19.1 % in the patients who received haploidentical HSCT and 44.8 % in those who received HLA-matched HSCT. The relapse rate of the patients who received HLA-matched HSCT appeared to be higher in our study than in previously published reports [5, 18, 28]. This difference might be due to one or a combination of the following factors. First, more patients transplanted in >CR1 were included in our study compared with other studies [5, 28]. Our results showed that >CR1 at transplantation was associated with an increased risk of relapse. Chen et al. also suggested that the remission status at the time of HSCT is significantly predictive of both LFS and OS [5]. Allo-HSCT in CR1 remains the standard of care at most centers [28]. Second, we included not only hematological and extramedullary relapse but also molecular relapse in our study. The patients in our study also did not receive “upfront” imatinib post-allogeneic HSCT, and imatinib treatment was only initiated if the BCR-ABL fusion product or extramedullary relapse was detected. Pfeifer et al. observed molecular recurrence in 56 % of patients overall [29]. There is insufficient evidence to date indicating that imatinib should be given to all patients following allogeneic HSCT [5]. Certain centers have reported that imatinib is poorly tolerated following myeloablative allogeneic HSCT [5, 17, 30]. A small study conducted at the University of Minnesota [20] showed a trend toward an improved outcome in patients treated with imatinib during the pre- and posttransplantation periods. Administration of imatinib maintenance therapy after HSCT has also been suggested to reduce the relapse rate and improve LFS in Ph+ ALL patients [5]. The only randomized study of the use of TKIs after allogeneic HSCT is currently being conducted by the GMALL group [29]. This study showed that prophylactic imatinib treatment significantly reduced the incidence of molecular relapse after allogeneic HSCT but that durable PCR negativity could still be achieved in the majority of posttransplantation patients with molecular relapse by restarting imatinib therapy. OS was found to significantly differ between the two treatment arms. Although haploidentical HSCT reduces the risk of disease recurrence, our study found no difference in either LFS or OS, possibly due to the small number of included patients and the beneficial effect of restarting imatinib in most patients.

In conclusion, we found a significant reduction in the relapse rate in Ph+ ALL patients who have received haploidentical HSCT compared with patients who have undergone HLA-matched HSCT. The incidences of aGVHD, cGVHD, and CMV viremia were higher in the patients who received haploidentical HSCT than in those who received HLA-matched HSCT, but there was no difference in NRM between these two groups. Although a well-designed, larger prospective study is needed to define the role of haploidentical HSCT in disease progression in patients with Ph+ ALL, our results suggest that haploidentical HSCT is a promising option for Ph+ ALL patients who lack a suitable HLA-matched donor.

## Abbreviations

aGVHD: acute graft-versus-host disease
allo-HSCT: allogeneic hematopoietic stem cell transplantation
ANC: absolute neutrophil count
Ara-C: arabinosylcytosine
ATG: anti-thymocyte globulin
BM: bone marrow
Bu: busulfan
cGVHD: chronic graft-versus-host disease
CMV: cytomegalovirus
CR: complete remission
CR1: first complete remission
CsA: cyclosporin
CY: cyclophosphamide
FB: fludarabine and busulfan
FK506: tacrolimus
G-BM: mobilize stem cells in the bone marrow
G-CSF: granulocyte colony stimulating factor
G-PB: mobilize stem cells in the peripheral blood
GVHD: graft-versus-host disease
GVL: graft-versus-leukemia
HRQoL: health-related quality of life
HSCT: hematopoietic stem cell transplantation
HVOD: hepatic venous occlusive disease
LFS: leukemia-free survival
MMF: mycophenolate mofetil
MNCs: mononuclear cells
MTX: methotrexate
NRM: non-relapse mortality
OS: overall survival
PB: peripheral blood
PBMC: peripheral blood mononuclear cell
Ph+ ALL: Philadelphia chromosome-positive acute lymphoblastic leukemia
qRT-PCR: real-time quantitative reverse transcription polymerase chain reaction
TBI: total-body irradiation
TKI: tyrosine kinase inhibitors
TRT: transplantation-related toxicity
WBC: white blood cell
